# ECOLOGICAL MOMENTARY ASSESSMENT TO MEASURE SOCIAL CONNECTEDNESS IN OLDER ADULTS: AN INTEGRATIVE REVIEW

**DOI:** 10.1093/geroni/igae098.3406

**Published:** 2024-12-31

**Authors:** JiYeon Choi, Seongmi Choi, Hun Kang, Jiyoung Shin

**Affiliations:** Yonsei University College of Nursing, Mo-Im Kim Nursing Research Institute, Seoul, Republic of Korea; Yonsei University College of Nursing, Mo-Im Kim Nursing Research Institute, Seoul, Republic of Korea; Yale University School of Public Health, New Haven, Connecticut, United States; Yonsei University, Seoul, Republic of Korea

## Abstract

While the importance of social connectedness in the lives of older adults is well-established, more comprehensive evidence is needed regarding its real-world operation. This study utilizes Ecological Momentary Assessment (EMA), increasingly employed in social and health sciences, to capture real-time social interactions in everyday environments, aiming to conduct an integrative review to understand multifactorial components of social connectedness and identify implementation strategies for older adults. Five databases (PubMed, Embase, Web of Science, CINAHL, and PsycINFO) were searched to retrieve studies published up to the year 2023. Of the 2,208 studies identified, 21 were selected for final analysis. Our analysis focused on identifying the multifactorial components of social connectedness (structural, functional, and quality) and assessment of EMA methods and participant adherence to the EMA. The components of social connectedness using EMA included the following: structural components such as social contacts or interactions (n=18), loneliness (n=9) as a functional component, and quality components, such as quality of social interactions (n=3). Additionally, as a context for understanding social connectedness, location at the time of assessment, and physical (e.g., pain, fatigue) and psychological states (e.g., anxiety, depression) were evaluated. Data were mostly collected using an app on digital devices (e.g., smartphone), with assessments conducted 5-6 times per day for 5 to 21 days, achieving an adherence rate of over 70%. Findings of this review highlight the current state of science in measuring social connectedness in older adults through EMA, focusing on its structural, functional, and quality components, and indicating its feasibility in real-world contexts.

